# Fisher Information Perspective of Pauli’s Electron †

**DOI:** 10.3390/e24121721

**Published:** 2022-11-24

**Authors:** Asher Yahalom

**Affiliations:** 1 Department of Electrical & Electronic Engineering, Faculty of Engineering, Ariel University, Ariel 40700, Israel; asya@ariel.ac.il; Tel.: +972-54-7740294; 2 Center for Astrophysics, Geophysics, and Space Sciences (AGASS), Ariel University, Ariel 40700, Israel

**Keywords:** spin, fluid dynamics, electromagnetic interaction

## Abstract

An electron moving at velocities much lower that the speed of light with a spin, is described by a wave function which is a solution of Pauli’s equation. It has been demonstrated that this system can be viewed as a vortical fluid which has remarkable similarities but also differences with classical ideal flows. In this respect, it was shown that the internal energy of the Pauli fluid can be interpreted, to some degree, as Fisher Information. In previous work on this subject, electromagnetic fields which are represented by a vector potential were ignored, here we remove this limitation and study the system under general electromagnetic interaction.

## 1. Introduction

Quantum mechanics is usually interpreted by the Copenhagen school approach. The Copenhagen approach defies the ontology of the quantum wave function and declares it to be completely epistemological (a tool for estimating probability of certain measurements) in accordance with the Kantian [1] conception of reality, and its denial of the human ability to grasp any thing “as it is” (ontology). However, historically we also see the development of another school of prominent scholars that interpret quantum mechanics quite differently. This school believed in the reality of the wave function. In their view, the wave function is part of reality in a similar sense as an electromagnetic field is. This approach, that was supported by Einstein and Bohm [2,3,4], has resulted in other interpretations of quantum mechanics; among them is the fluid realization championed by Madelung [5,6], which stated that the modulus square of the wave function is a fluid density and the phase is a potential of the velocity field of the fluid. More recently quantum macroscopic models were derived from a Wigner–Boltzmann equation using a diffusion scaling. It was shown in analogy to the semi-classical situation, that quantum hydrodynamic models can be derived by employing a hydrodynamic scaling [7]. However, this approach was constrained to wave functions of spinless electrons and could not take into account a complete set of attributes even for slow moving (with respect to the speed of light) electrons.

A non-relativistic quantum equation for a spinor was first introduced by Wolfgang Pauli in 1927 [8]. This equation is based on a two-dimensional operator Hamiltonian matrix. The two-dimensional operator Hamiltonians matrices are currently abundant in the literature ([9,10,11,12,13,14,15,16,17,18,19,20,21,22]) and describe many types of quantum systems. It is natural to inquire whether such a theory can be given a fluid dynamical interpretation. This question is of great importance as supporters of the non-realistic Copenhagen school of quantum mechanics usually use the spin concept as a proof that nature is inherently quantum and, thus, has elements without classical analogue or interpretation. A Bohmian analysis of the Pauli equation was given by Holland and others [3], however, the analogy of the Pauli theory to fluid dynamics and the notion of spin vorticity were not considered. This state of affairs was corrected in [23] for an ensemble of particles based on the Wigner approach and was applied to Graphene. In [24], spin fluid dynamics was introduced for a single electron with a spin. Here, we mention that a kinetic approach, which can, in principle, lead to a fluid formalism, was suggested by [25], in which an approach to the spin-state description by means of the probability distributions of dichotomic random variables was introduced.

The interpretation of Pauli’s spinor in terms of fluid density and velocity variables leads us directly to the nineteenth century seminal work of Clebsch [26,27], which is strongly related to the variational analysis of fluids. Variational principles for barotropic fluid dynamics are described in the literature. A four function variational principle for an Eulerian barotropic fluid was depicted by Clebsch [26,27] and much later by Davidov [28] whose main purpose was to quantize fluid dynamics. The work was written in Russian, and was largely unknown in the West. Lagrangian fluid dynamics (which takes a different approach than Eulerian fluid dynamics) was given a variational description by Eckart [29]. Ignoring both the work of Clebsch (written in German) and the work of Davidov (written in Russian) initial attempts in the English written literature to formulate Eulerian fluid dynamics using a variational principle, were given by Herivel [30], Serrin [31], and Lin [32]. However, the variational principles developed by the above authors were cumbersome, relying on quite a few “Lagrange multipliers” and auxiliary “potentials”. The total number of independent functions in the above formulations are from 11 to 7, which are much more than the four functions required for the Eulerian and continuity equations of a barotropic flow. Thus, those methods did not have practical use. Seliger and Whitham [33] have reintroduced the variational formalism of Clebsch depending on only four variables for barotropic flow. Lynden-Bell and Katz [34] have described a variational principle in terms of two functions the load λ and density ρ. However, their formalism contains an implicit definition for the velocity $\vec{v}$, such that one is required to solve a partial differential equation in order to obtain both $\vec{v}$ in terms of ρ and λ, as well as its variations. Much the same criticism holds for their general variational for non-barotropic flows [35]. Yahalom and Lynden-Bell [36] overcame the implicity definition limitation by paying the price of adding an additional single variational variable. This formalism allows arbitrary variations (not constrained) and the definition of $\vec{v}$ is explicit.

A fundamental issue in the fluid interpretation of quantum mechanics still remains. This refers to the meaning of thermodynamic quantities. Thermodynamics concepts, such as specific enthalpy, pressure, and temperature are related to the specific internal energy defined by the equation of state as a unique function of entropy and density. The internal energy is a part of any Lagrangian density related to fluid dynamics. The internal energy functional can, in principle, be explained on the basis of the microscopic composition of the fluid using statistical physics. That is the atoms and molecules from which the fluid is composed and their interactions impose an equation of state. However, a quantum fluid has no structure and yet the equations of both the spinless [5,6] and spin [24] quantum fluid dynamics shows that terms analogue to internal energies appear. One, thus, is forced to inquire where do those internal energies originate? Of course one cannot suggest that the quantum fluid has a microscopic sub-structure as this will defy current empirical evidence, suggesting that the electron is a point particle. The answer to this question comes from an entirely different scientific discipline known as measurement theory [37,38]. Fisher information is a basic notion of measurement theory, and is a figure of merit of a measurement quality of any quantity. It was demonstrated [38] that this notion is the internal energy of a spinless electron (up to a proportionality constant) and can be used to partially interpret the internal energy of an electron with spin. Here, we should mention an attempt to derive most physical theories from Fisher information as described by Frieden [39]. It was suggested [40] that there exist a velocity field such that the Fisher information will be given a complete explanation for the spin fluid internal energy. It was also suggested that one may define comoving scalar fields as in ideal fluid mechanics, however, this was only demonstrated implicitly but not explicitly. A common feature of previous work on the fluid and Fisher information interpretation of quantum mechanics, is the negligence of electromagnetic interaction thus setting the vector potential to zero. This makes sense as the classical ideal fluids discussed in the literature are not charged. Hence, in order to make the comparison easier to comprehend the vector potential should be neglected. However, one cannot claim a complete description of quantum mechanics lacking a vector potential thus ignoring important quantum phenomena, such as the Zeeman effect, which depends on a vector potential through the magnetic field.

We will begin this paper by introducing the Eulerian–Clebsch variational principle for a charged fluid (A variational principle for a charged classical particle with a vector potential interaction, a system of the same, and the Eckart [29] Lagrangian variational principles generalized for a charged fluid are given in the Appendix A and Appendix B). Next, we will introduce the Fisher information and the concept of probability amplitude. This will be followed by a discussion of Schrödinger equation with a non-trivial vector potential and its interpretation in terms of Madelung fluid dynamics. The variational principle of a charged Madelung fluid dynamics will be described and its relations to Fisher information will be elucidated. Then, we introduce Pauli’s equation with a vector potential and interpret it in terms of generalized Clebsch variables and discuss the similarities and differences between a charged Clebsch flow and spin fluid dynamics and their variational principles. Finally, the concept of Fisher information will be introduced in the framework of spin fluid dynamics.

## 2. An Eulerian Charged Fluid—The Clebsch Approach

In this section, we follow closely the analysis of [24] with the modification of taking into account the electromagnetic interaction, which was neglected in our previous work. Consider the action:

(1) $$\begin{array}{ccc} A & \equiv & \int Ld^{3}xdt,\;\;\;\;\;L\equiv L_{0}+L_{2}+L_{i}\\ L_{0} & \equiv & \rho \left(\frac{1}{2}v^{2}-\varepsilon \right),\;L_{2}\equiv \nu \left[\frac{\partial \rho }{\partial t}+\vec{\nabla }\cdot \left(\rho \vec{v}\right)\right]-\rho \alpha \frac{d\beta }{dt}\\ L_{i} & = & \rho_{c}\left(\vec{A}\cdot \vec{v}-\varphi \right) \end{array}$$

In the Eulerian approach, we consider the variational variables to be fields, that are functions of space and time. We have two such variational variables the vector velocity field $\vec{v}\left(\vec{x},t\right)$ and density scalar field $\rho (\vec{x},t)$. The conservation of quantities, such as the label of the fluid element, mass, charge, and entropy are dealt by introducing Lagrange multipliers ν,α in such a way that the variational principle will yield the following equations:

(2) $$\begin{array}{ccc} & & \frac{\partial \rho }{\partial t}+\vec{\nabla }\cdot \left(\rho \vec{v}\right)=0\\ & & \frac{d\beta }{dt}=0 \end{array}$$

Provided ρ is not null those are just the continuity equation which ensures mass conservation and the conditions that β is comoving and is, thus, a label, further analysis to be described below shows that α is a label too. This is why in the Eulerian approach we are obliged to add the Lagrangian density $L_{2}$. The specific internal energy ε defined in Equation (A24) is dependent on the thermodynamic properties of the specific fluid. It generally depends, through a given “equation of state”, on the density and specific entropy. In our case, we shall assume a barotropic fluid, that is a fluid in which ε(ρ) is a function of the density ρ only. Other functions connected to the electromagnetic interaction, such as the potentials $\vec{A},\varphi$ are assumed given function of coordinates and are not varied. Another simplification which we introduce is the assumption that the fluid element is made of microscopic particles having a given mass *m* and a charge *e*, in this case it follows from Equation (A57) that:

(3) $$\rho_{c}=k\rho .$$

Let us take an arbitrary variational derivative of the above action with respect to $\vec{v}$, this will result in:

(4) $$\begin{array}{ccc} \delta_{\vec{v}}A & = & \int d^{3}xdt\rho \delta \vec{v}\cdot \left[\vec{v}-\vec{\nabla }\nu -\alpha \vec{\nabla }\beta +k\vec{A}\right]\\ & + & \oint d\vec{S}\cdot \delta \vec{v}\rho \nu +\int d\vec{\Sigma }\cdot \delta \vec{v}\rho \left[\nu \right], \end{array}$$

the above boundary terms contain integration over the external boundary $\oint d\vec{S}$ and an integral over the cut $\int d\vec{\Sigma }$ that must be introduced in case that ν is not single valued. The external boundary term vanishes; in the case of astrophysical flows, for which ρ=0 on the free flow boundary, or the case in which the fluid is contained in a vessel which induces a no flux boundary condition $\delta \vec{v}\cdot \hat{n}=0$ ($\hat{n}$ is a unit vector normal to the boundary). The cut “boundary” term vanish when the velocity field varies only parallel to the cut that is it satisfies a Kutta type condition. If the boundary terms vanish, $\vec{v}$ must have the following form:

(5) $$\vec{v}=\hat{\vec{v}}\equiv \alpha \vec{\nabla }\beta +\vec{\nabla }\nu -k\vec{A}$$

this is a generalization of Clebsch representation of the flow field (see for example [29], (page 248, [41])) for a charged flow. The vorticity of such a flow is:

(6) $$\vec{\omega }=\vec{\nabla }\times \vec{v}=\vec{\nabla }\alpha \times \vec{\nabla }\beta -k\vec{B}$$

in which we have taken into account the definition of the magnetic field given in Equation (A6). Let us now take the variational derivative with respect to the density ρ, we obtain:

(7) $$\begin{array}{ccc} \delta_{\rho }A & = & \int d^{3}xdt\delta \rho \left[\frac{1}{2}{\vec{v}}^{2}-w-\frac{\partial \nu }{\partial t}-\vec{v}\cdot \vec{\nabla }\nu +k\left(\vec{A}\cdot \vec{v}-\varphi \right)\right]\\ & + & \oint d\vec{S}\cdot \vec{v}\delta \rho \nu +\int d\vec{\Sigma }\cdot \vec{v}\delta \rho \left[\nu \right]+\int d^{3}{x\nu \delta \rho |}_{t_{0}}^{t_{1}} \end{array}$$

in which $w=\frac{\partial (\rho \varepsilon )}{\partial \rho }$ is the specific enthalpy (see Equation (A33)). Hence, provided that δρ vanishes on the boundary of the domain, on the cut and in initial and final times the following equation must be satisfied:

(8) $$\frac{d\nu }{dt}=\frac{\partial \nu }{\partial t}+\vec{v}\cdot \vec{\nabla }\nu =\frac{1}{2}{\vec{v}}^{2}-w+k\left(\vec{A}\cdot \vec{v}-\varphi \right)$$

In the above, we notice that taking a time derivative for a fixed label $\vec{\alpha }$ (also known as a material derivative) of any quantity *g* takes the form:

(9) $$\frac{dg(\vec{\alpha },t)}{dt}=\frac{dg(\vec{x}\left(\vec{\alpha },t\right),t)}{dt}=\frac{\partial g}{\partial t}+\frac{d\vec{x}}{dt}\cdot \vec{\nabla }g=\frac{\partial g}{\partial t}+\vec{v}\cdot \vec{\nabla }g$$

once *g* is considered to be a field dependent on $\vec{x},t$. Finally we have to calculate the variation with respect to β, this will lead us to the following results:

(10) $$\begin{array}{ccc} \delta_{\beta }A & = & \int d^{3}xdt\delta \beta \left[\frac{\partial \left(\rho \alpha \right)}{\partial t}+\vec{\nabla }\cdot \left(\rho \alpha \vec{v}\right)\right]\\ & - & \oint d\vec{S}\cdot \vec{v}\rho \alpha \delta \beta -\int d\vec{\Sigma }\cdot \vec{v}\rho \alpha \left[\delta \beta \right]-\int d^{3}{x\rho \alpha \delta \beta |}_{t_{0}}^{t_{1}} \end{array}$$

Hence, choosing δβ in such a way that the temporal and spatial boundary terms vanish (this includes choosing δβ to be continuous on the cut if one needs to introduce such a cut) in the above integral will lead to the equation:

(11) $$\frac{\partial \left(\rho \alpha \right)}{\partial t}+\vec{\nabla }\cdot \left(\rho \alpha \vec{v}\right)=0$$

Using the continuity Equation (2) this will lead to the equation:

(12) $$\frac{d\alpha }{dt}=0$$

Hence, for ρ≠0, both α and β are comoving coordinates.

### 2.1. Euler’s Equations

We shall now show that a velocity field given by Equation (5), such that the functions α,β,ν satisfy the corresponding Equations (2), (8) and (12) must satisfy Euler’s equations. Let us calculate the material derivative of $\vec{v}$:

(13) $$\frac{d\vec{v}}{dt}=\frac{d\vec{\nabla }\nu }{dt}+\frac{d\alpha }{dt}\vec{\nabla }\beta +\alpha \frac{d\vec{\nabla }\beta }{dt}-k\frac{d\vec{A}}{dt}$$

It can be easily shown that:

(14) $$\begin{array}{ccc} \frac{d\vec{\nabla }\nu }{dt} & = & \vec{\nabla }\frac{d\nu }{dt}-\vec{\nabla }v_{n}\frac{\partial \nu }{\partial x_{n}}=\vec{\nabla }\left(\frac{1}{2}{\vec{v}}^{2}-w+k\vec{A}\cdot \vec{v}-k\varphi \right)-\vec{\nabla }v_{n}\frac{\partial \nu }{\partial x_{n}}\\ \frac{d\vec{\nabla }\beta }{dt} & = & \vec{\nabla }\frac{d\beta }{dt}-\vec{\nabla }v_{n}\frac{\partial \beta }{\partial x_{n}}=-\vec{\nabla }v_{n}\frac{\partial \beta }{\partial x_{n}} \end{array}$$

In which $x_{n}$ is a Cartesian coordinate and a summation convention is assumed. Inserting the result from Equations (14) into Equation (13) yields:

(15) $$\begin{array}{ccc} & & \frac{d\vec{v}}{dt}=-\vec{\nabla }v_{n}\left(\frac{\partial \nu }{\partial x_{n}}+\alpha \frac{\partial \beta }{\partial x_{n}}\right)+\vec{\nabla }\left(\frac{1}{2}{\vec{v}}^{2}-w+k\vec{A}\cdot \vec{v}-k\varphi \right)-k\frac{d\vec{A}}{dt}\\ & = & -\vec{\nabla }v_{n}\left(v_{n}+kA_{n}\right)+\vec{\nabla }\left(\frac{1}{2}{\vec{v}}^{2}-w+k\vec{A}\cdot \vec{v}-k\varphi \right)-k\partial_{t}\vec{A}-k\left(\vec{v}\cdot \vec{\nabla }\right)\vec{A}\\ & = & -\vec{\nabla }w+k\vec{E}+k\left(v_{n}\vec{\nabla }A_{n}-v_{n}\partial_{n}\vec{A}\right), \end{array}$$

in the above we have used the electric field defined in Equation (A6). We notice that according to Equation (A7):

(16) $${\left(v_{n}\vec{\nabla }A_{n}-v_{n}\partial_{n}\vec{A}\right)}_{l}=v_{n}\left(\partial_{l}A_{n}-\partial_{n}A_{l}\right)=\epsilon_{lnj}v_{n}B_{j}={\left(\vec{v}\times \vec{B}\right)}_{l},$$

Hence, we obtain the Euler equation of a charged fluid in the form:

(17) $$\frac{d\vec{v}}{dt}=-\vec{\nabla }w+k\left[\vec{v}\times \vec{B}+\vec{E}\right]=-\frac{1}{\rho }\vec{\nabla }P+k\left[\vec{v}\times \vec{B}+\vec{E}\right],$$

since in a barotropic fluid (see Equation (A34)):

(18) $$\vec{\nabla }w=\frac{\partial w}{\partial \rho }\vec{\nabla }\rho =\frac{1}{\rho }\frac{\partial P}{\partial \rho }\vec{\nabla }\rho =\frac{1}{\rho }\vec{\nabla }P.$$

The above equation is identical to Equation (A56) and, thus, proves that the Euler equations can be derived from the action given in Equation (2) and, hence, all the equations of charged fluid dynamics can be derived from the above action without restricting the variations in any way.

### 2.2. Simplified Action

The reader of this paper might argue that the authors have introduced unnecessary complications to the theory of fluid dynamics by adding three more functions α,β,ν to the standard set $\vec{v},\rho$. In the following, we will show that this is not so and the action given in Equation (2) in a form suitable for a pedagogic presentation can indeed be simplified. It is easy to show that the Lagrangian density appearing in Equation (2) can be written in the form:

(19) $$\begin{array}{ccc} L & = & -\rho \left[\frac{\partial \nu }{\partial t}+\alpha \frac{\partial \beta }{\partial t}+\varepsilon \left(\rho \right)+k\varphi \right]+\frac{1}{2}\rho \left[{\left(\vec{v}-\hat{\vec{v}}\right)}^{2}-{\hat{\vec{v}}}^{2}\right]\\ & + & \frac{\partial \left(\nu \rho \right)}{\partial t}+\vec{\nabla }\cdot \left(\nu \rho \vec{v}\right) \end{array}$$

In which $\hat{\vec{v}}$ is a shorthand notation for $\vec{\nabla }\nu +\alpha \vec{\nabla }\beta -k\vec{A}$ (see Equation (5)). Thus L has three contributions:

(20) $$\begin{array}{ccc} L & = & \hat{L}+L_{\vec{v}}+L_{boundary}\\ \hat{L} & \equiv & -\rho [\frac{\partial \nu }{\partial t}+\alpha \frac{\partial \beta }{\partial t}+\varepsilon \left(\rho \right)+k\varphi +\frac{1}{2}{\left(\vec{\nabla }\nu +\alpha \vec{\nabla }\beta -k\vec{A}\right)}^{2}]\\ L_{\vec{v}} & \equiv & \frac{1}{2}\rho {\left(\vec{v}-\hat{\vec{v}}\right)}^{2}\\ L_{boundary} & \equiv & \frac{\partial \left(\nu \rho \right)}{\partial t}+\vec{\nabla }\cdot \left(\nu \rho \vec{v}\right) \end{array}$$

The only term containing $\vec{v}$ is $L_{\vec{v}}$, it can easily be seen that this term will lead, after we nullify the variational derivative, to Equation (5) but will otherwise have no contribution to other variational derivatives. Notice that the term $L_{boundary}$ contains only complete partial derivatives and, thus, cannot contribute to the equations although it can change the boundary conditions. Hence, we see that Equations (2), (8) and (12) can be derived using the Lagrangian density $\hat{L}$ in which $\hat{\vec{v}}$ replaces $\vec{v}$ in the relevant equations. Furthermore, after integrating the four Equations (2), (8) and (12) we can insert the potentials α,β,ν into Equation (5) to obtain the physical velocity $\vec{v}$. Hence, the general barotropic fluid dynamics problem is changed such that instead of solving the Euler and continuity equations we need to solve an alternative set which can be derived from the Lagrangian density $\hat{L}$.

The Lagrangian density $\hat{L}$ has two manifest properties that will repeat in both Schrodinger and Pauli quantum mechanics, but are not evident in other formulations of classical mechanics. First, it contains a term quadratic in the vector potential $\vec{A}$, this cannot be found in classical Lagrangians which are described in previous sections, in those cases the Lagrangians are always linear in $\vec{A}$. Second, the gauge freedom described in Equation (A15) is preserved by redefining the variation variable:

(21) $$\nu^{\prime }=\nu -k\Lambda .$$

However, before we discuss quantum theory a remark on Fisher information is required.

## 3. Fisher Information

Let there be a random variable *X* with probability density function (PDF) $f_{X}\left(x\right)$. The Fisher Information for a PDF which is translationaly invariant is given by the form:

(22) $$F_{I}=\int dx{\left(\frac{df_{X}}{dx}\right)}^{2}\frac{1}{f_{X}}$$

It was shown [37,39] that the standard deviation $\sigma_{X}$ of any random variable is bounded from below, such that:

(23) $$\sigma_{X}\geq \sigma_{Xmin}=\frac{1}{F_{I}}$$

Hence, the higher the Fisher information we have about the variable, the smaller standard deviation we may achieve and, thus, our knowledge about the value of this random variable is greater. This is known as the Cramer Rao inequality. Fisher information is most elegantly introduced in terms of the probability amplitude:

(24) $$f_{X}=a^{2}\Rightarrow F_{I}=4\int dx{\left(\frac{da}{dx}\right)}^{2}$$

In this work, we will be interested in a three-dimensional random variable designating the position of an electron, hence:

(25) $$F_{I}=\int d^{3}x{\left(\vec{\nabla }f_{\vec{X}}\right)}^{2}\frac{1}{f_{\vec{X}}}=4\int d^{3}x{\left(\vec{\nabla }a\right)}^{2}\equiv \int d^{3}xF_{I}$$

In the above, $F_{I}\equiv 4{\left(\vec{\nabla }a\right)}^{2}$ is the Fisher information density. The Fisher information is the only intuitive reason to consider a “probability density amplitude” a notion which is quite important in quantum mechanics as it is also the amplitude of the quantum wave function to be described in the next section.

## 4. Schrödinger’s Theory Formulated in Terms of Fluid Mechanics

### 4.1. Background

Quantum mechanics has lost faith in our ability to predict precisely the whereabouts of even a single particle. What the theory does predict precisely is the evolution in time of a quantity denoted “the quantum wave function”, which is related to a quantum particle whereabouts in a statistical manner. This evolution is described by an equation suggested by Schrödinger [42]:

(26) $$i\hbar \dot{\psi }=\hat{H}\psi ,\;\hat{H}=-\frac{1}{2m}{\left(\hbar \vec{\nabla }-ie\vec{A}\right)}^{2}+e\varphi$$

in the above $i=\sqrt{-1}$ and ψ is the complex wave function. $\dot{\psi }=\frac{\partial \psi }{\partial t}$ is the partial time derivative of the wave function. $\hbar =\frac{h}{2\pi }$ is Planck’s constant divided by 2π and *m* is the particles mass. The Lagrangian density L for the non-relativistic electron is written as:

(27) $$L_{S}=\frac{1}{2}i\hbar \left(\psi^{*}\dot{\psi }-\dot{\psi^{*}}\psi \right)-\psi^{*}\hat{H}\psi$$

A straightforward variation of the action:

(28) $$A_{S}\equiv \int_{t1}^{t2}L_{S}dt\equiv \int_{t1}^{t2}\int L_{S}d^{3}xdt$$

with respect to $\psi^{*}$ will lead to Equation (26) (while a variation with respect to ψ will lead to a complex conjugate of the same). However, this presentation of quantum mechanics is rather abstract and does not give any physical picture regarding the meaning of the quantities involved. Thus, we write the quantum wave function using its modulus *a* and phase ϕ:

(29) $$\psi =ae^{i\phi }$$

the Lagrangian density takes the form:

(30) $$L_{S}=-\frac{\hbar^{2}}{2m}\left[{\left(\vec{\nabla }a\right)}^{2}+a^{2}{\left(\vec{\nabla }\phi \right)}^{2}\right]-ea^{2}\varphi -\hbar a^{2}\frac{\partial \phi }{\partial t}+ea^{2}\vec{A}\cdot {\vec{v}}_{S}+\frac{e^{2}}{2m}a^{2}A^{2}$$

in we define:

(31) $${\vec{v}}_{S}=\frac{\hbar }{m}\vec{\nabla }\phi -\frac{e}{m}\vec{A}$$

The variational derivative of this with respect to ϕ yields the continuity equation:

(32) $$\frac{\delta A_{S}}{\delta \phi }=0\to \frac{\partial \hat{\rho }}{\partial t}+\vec{\nabla }\cdot \left(\hat{\rho }{\vec{v}}_{S}\right)=0$$

in which the mass density is defined as:

(33) $$\hat{\rho }=ma^{2}.$$

Hence, ${\vec{v}}_{S}$ field is the velocity associated with the probability, charge, and mass flow. Variationally deriving with respect to *a* leads to the Hamilton Jacobi equation:

(34) $$\frac{\delta A_{S}}{\delta a}=0\to \frac{\partial S}{\partial t}+\frac{1}{2m}{\left(\vec{\nabla }S-e\vec{A}\right)}^{2}+e\varphi =\frac{\hbar^{2}\nabla^{2}a}{2ma}$$

in which: S=ℏϕ. The right-hand side of the above equation contains the “quantum correction”. These results are elementary, but their derivation illustrates the advantages of using the two variables, phase and modulus, to obtain equations of motion that have a substantially different form than the familiar Schrödinger equation (although having the same mathematical content) and have straightforward physical interpretations [2]. The interpretation is, of course, connected to the modulus being a physical observable (by Born’s interpretational postulate) and to the phase having a similar though somewhat more problematic status (The “observability” of the phase has been discussed in the literature by various sources, e.g., in [43] and, in connection with a recent development, in [11,13]).

### 4.2. Similarities between Potential Fluid Dynamics and Quantum Mechanics

In writing the Lagrangian density of quantum mechanics in the modulus-phase representation, Equation (30), one notices a striking similarity between this Lagrangian density and that of potential fluid dynamics (fluid dynamics without vorticity), as represented in the work of Clebsch [33]. The connection between fluid dynamics and quantum mechanics of an electron was already discussed by Madelung [5] and in Holland’s book [3]. However, the discussion by Madelung refers to the equations only, and does not address the variational formalism which was discussed in [24] and is repeated here with the important addition of an electromagnetic interaction.

If a flow satisfies the condition of zero vorticity in the absence of a magnetic field, i.e., the velocity field $\vec{v}$ is such that $\vec{\nabla }\times \vec{v}=0$, then there exists a function ν, such that $\vec{v}=\vec{\nabla }\nu$. The above statement is equivalent to taking a Clebsch representation of the velocity field but with α=β=0. In that case, following Equation (20), one can describe the fluid mechanical system with the following Lagrangian density:

(35) $$\hat{L}=-\left[\frac{\partial \nu }{\partial t}+\frac{1}{2}{\left(\vec{\nabla }\nu -k\vec{A}\right)}^{2}+\varepsilon \left(\rho \right)+k\varphi \right]\rho$$

by inserting α=β=0 in $\hat{L}$ [38]. Taking the variational derivative with respect to ν and ρ, one obtains the following equations:

(36) $$\begin{array}{ccc} \frac{\partial \rho }{\partial t} & + & \vec{\nabla }\cdot \left(\rho \left(\vec{\nabla }\nu -k\vec{A}\right)\right)=0 \end{array}$$

(37) $$\begin{array}{ccc} \frac{\partial \nu }{\partial t} & = & -\frac{1}{2}{\left(\vec{\nabla }\nu -k\vec{A}\right)}^{2}-w-k\varphi . \end{array}$$

The first of those equations is the continuity equation, while the second is Bernoulli’s equation.

Going back to the quantum mechanical system described by Equation (30), we introduce the following variable: $\hat{\nu }=\frac{\hbar \phi }{m}=\frac{S}{m}$. In terms of these new variables, the Lagrangian density in Equation (30) will take the form:

(38) $$L_{S}=-\left[\frac{\partial \hat{\nu }}{\partial t}+\frac{1}{2}{\left(\vec{\nabla }\hat{\nu }-k\vec{A}\right)}^{2}+\frac{\hbar^{2}}{2m^{2}}\frac{{\left(\vec{\nabla }\sqrt{\hat{\rho }}\right)}^{2}}{\hat{\rho }}+k\varphi \right]\hat{\rho }$$

When compared with Equation (35) the following correspondence is noted:

(39) $$\hat{\nu }\Leftrightarrow \nu ,\;\hat{\rho }\Leftrightarrow \rho ,\;\frac{\hbar^{2}}{2m^{2}}\frac{{\left(\vec{\nabla }\sqrt{\hat{\rho }}\right)}^{2}}{\hat{\rho }}\Leftrightarrow \varepsilon .$$

The quantum “internal energy”:

(40) $$\varepsilon_{q}\equiv \frac{\hbar^{2}}{2m^{2}}\frac{{\left(\vec{\nabla }\sqrt{\hat{\rho }}\right)}^{2}}{\hat{\rho }}$$

depends also on the derivative of the density and in this sense it is non-local. This is unlike the fluid case, in which internal energy is a function of the mass density only. However, in both cases, the internal energy is a positive quantity. Unlike classical systems, in which the Lagrangian is quadratic in the time derivatives of the degrees of freedom (see Equation (A1)), the Lagrangians of both quantum and Eulerian fluid dynamics are linear in the time derivatives of the degrees of freedom. We also note that gauge freedom is preserved (see Equation (A15)) provided that the variational quantum potential $\hat{\nu }$ (or quantum phase) is redefined as:

(41) $${\hat{\nu }}^{\prime }=\hat{\nu }-k\Lambda ,\;\phi^{\prime }=\phi -\frac{e}{\hbar }\Lambda ,$$

this means that the global phase of a quantum wave function does not have a physical meaning, in contrast to the quantity ${\vec{v}}_{S}$ that does (see Equation (31)), as it is invariant under gauge transformations. Finally, we note that the concept of quantum internal energy is closely related to the concept of quantum potential [3] (see the right side of Equation (34)):

(42) $$Q=-\frac{\hbar^{2}}{2m}\frac{{\vec{\nabla }}^{2}\sqrt{\hat{\rho }}}{\sqrt{\hat{\rho }}}.$$

Additionally, also that in the limit ℏ→0 Schrödinger’s quantum mechanics is essentially a potential fluid flow without pressure or internal energy.

### 4.3. Madelung Flows in Terms of Fisher Information

As explained in the introduction, the quantum Madelung flow does not have a microstructure that will explain its internal energy. To understand the origins of this term let us look at the internal energy of Equation (38), the term appearing in the Lagrangian density has the form:

(43) $$\hat{\rho }\varepsilon_{q}=\frac{\hbar^{2}}{2m^{2}}{\left(\vec{\nabla }\sqrt{\hat{\rho }}\right)}^{2}=\frac{\hbar^{2}}{2m}{\left(\vec{\nabla }a\right)}^{2}.$$

Comparing this to Equation (25) we arrive at the result:

(44) $$\hat{\rho }\varepsilon_{q}=\frac{\hbar^{2}}{8m}F_{Iq}.\;F_{Iq}\equiv 4{\left(\vec{\nabla }a\right)}^{2}$$

Thus, the Lagrangian density of the Madelung flow can be written as:

(45) $$L=-\left[\frac{\partial \hat{\nu }}{\partial t}+\frac{1}{2}{\left(\vec{\nabla }\hat{\nu }-k\vec{A}\right)}^{2}+k\varphi \right]\hat{\rho }-\frac{\hbar^{2}}{8m}F_{Iq}$$

The pre-factor $\frac{\hbar^{2}}{8m}$ seems to appear in every case in which Fisher information appears in a quantum Lagrangian and may be significant. This is the case, also, in spin fluid dynamics, as will be shown in the next section.

Thus, Schrödinger’s fluid differ from a classical potential flow in that it takes into account Fisher information as a driving force, and its “fluid element” lacks an internal energy, that is, it is truly microscopic and has no structure. Thus, when written explicitly in terms of physical quantities and not mere abstractions, quantum mechanics reveals itself as a theory that takes into account information gain (or loss) as a force of nature. We conclude this section by quoting Anton Zeilinger’s recent remark to the press, that it is quantum mechanics that demonstrates that information is more fundamental than space-time.

## 5. Spin

Schrödinger’s quantum mechanics is limited to the description of spinless particles and its fluid dynamics representation is limited to zero vorticity (for the zero magnetic field case) potential flows. This suggests that a quantum theory of particles with spin may have a fluid dynamics representation which cannot be described solely by using $\hat{\nu }$ and requires the full Clebsch apparatus. The Pauli equation for a non-relativistic particle with spin is given by:

(46) $$i\hbar \dot{\psi }=\hat{H}\psi ,\;\hat{H}=-\frac{\hbar^{2}}{2m}{\left[\vec{\nabla }-\frac{ie}{\hbar }\vec{A}\right]}^{2}+\mu \vec{B}\cdot \vec{\sigma }+e\varphi$$

ψ here is a two-dimensional complex column vector (also denoted as spinor), $\hat{H}$ is a two-dimensional hermitian operator matrix, μ is the magnetic moment of the particle. $\vec{\sigma }$ is a vector of two-dimensional Pauli matrices which can be represented as follows:

(47) $$\sigma_{1}=\left(\begin{array}{cc} 0 & 1\\ 1 & 0 \end{array}\right),\;\sigma_{2}=\left(\begin{array}{cc} 0 & -i\\ i & 0 \end{array}\right),\;\sigma_{3}=\left(\begin{array}{cc} 1 & 0\\ 0 & -1 \end{array}\right)$$

A spinor ψ satisfying Equation (46) must also satisfy a continuity equation of the form:

(48) $$\frac{\partial \rho }{\partial t}+\vec{\nabla }\cdot \vec{j}=0.$$

In the above:

(49) $$\rho =\psi^{†}\psi ,\;\vec{j}=\frac{\hbar }{2mi}\left[\psi^{†}\vec{\nabla }\psi -\left(\vec{\nabla }\psi^{†}\right)\psi \right]-k\vec{A}\rho .$$

The symbol $\psi^{†}$ represents a row spinor (the transpose) whose components are equal to the complex conjugate of the column spinor ψ. Comparing the standard continuity equation to Equation (48) suggests the definition of a velocity field as follows [3]:

(50) $$\vec{v}=\frac{\vec{j}}{\rho }=\frac{\hbar }{2mi\rho }\left[\psi^{†}\vec{\nabla }\psi -\left(\vec{\nabla }\psi^{†}\right)\psi \right]-k\vec{A}.$$

A variational description of the Pauli system can be given using the following Lagrangian density:

(51) $$L=\frac{1}{2}i\hbar \left(\psi^{†}\dot{\psi }-\dot{\psi^{†}}\psi \right)-\psi^{†}\hat{H}\psi$$

Holland [3] has suggested the following representation of the spinor:

(52) $$\psi =Re^{i\frac{\chi }{2}}\left(\begin{array}{c} \cos\left(\frac{\theta }{2}\right)e^{i\frac{\phi }{2}}\\ i\sin\left(\frac{\theta }{2}\right)e^{-i\frac{\phi }{2}} \end{array}\right)\equiv \left(\begin{array}{c} \psi_{↑}\\ \psi_{↓} \end{array}\right).$$

In terms of this representation the density is given as:

(53) $$\rho =\psi^{†}\psi =R^{2}\Rightarrow R=\sqrt{\rho }.$$

The mass density is given as:

(54) $$\hat{\rho }=m\rho =m\psi^{†}\psi =mR^{2}.$$

The probability amplitudes for spin up and spin down electrons are given by:

(55) $$a_{↑}=\left|\psi_{↑}\right|=R\left|\cos\frac{\theta }{2}\right|,\;a_{↓}=\left|\psi_{↓}\right|=R\left|\sin\frac{\theta }{2}\right|$$

Let us now look at the expectation value of the spin:

(56) $$<\frac{\hbar }{2}\vec{\sigma }>=\frac{\hbar }{2}\int \psi^{†}\vec{\sigma }\psi d^{3}x=\frac{\hbar }{2}\int \left(\frac{\psi^{†}\vec{\sigma }\psi }{\rho }\right)\rho d^{3}x$$

The spin density can be calculated using the representation given in Equation (52) as:

(57) $$\hat{s}\equiv \frac{\psi^{†}\vec{\sigma }\psi }{\rho }=\left(\sin\theta \sin\phi ,\sin\theta \cos\phi ,\cos\theta \right)$$

This gives an easy physical interpretation to the variables θ,ϕ as angles which describe the projection of the spin density on the axes. θ is the elevation angle of the spin density vector and ϕ is the azimuthal angle of the same. The velocity field can now be calculated by inserting ψ given in Equation (52) into Equation (50):

(58) $$\vec{v}=\frac{\hbar }{2m}\left(\vec{\nabla }\chi +\cos\theta \vec{\nabla }\phi \right)-k\vec{A}.$$

Comparing Equation (58) with the generalized Clebsch form given in Equation (5) suggest the following identification:

(59) $$\alpha =\cos\theta ,\;\beta =\frac{\hbar }{2m}\phi ,\;\nu =\frac{\hbar }{2m}\chi .$$

Notice that α is single valued, but β and ν are not. Obviously, this velocity field will have a generically non-vanishing vorticity even if the magnetic field is null:

(60) $$\begin{array}{ccc} \vec{\omega } & = & \vec{\nabla }\times \vec{v}=\vec{\nabla }\alpha \times \vec{\nabla }\beta -k\vec{B}\\ & = & \frac{\hbar }{2m}\vec{\nabla }\cos\theta \times \vec{\nabla }\phi -k\vec{B}=\frac{\hbar }{2m}\sin\theta \vec{\nabla }\phi \times \vec{\nabla }\theta -k\vec{B}. \end{array}$$

We see again that the gauge symmetry (Equation (A15)) is maintained provided that the variational variable ν proportional to the global phase χ is redefined:

(61) $$\nu^{\prime }=\nu -k\Lambda .$$

Inserting the representation of ψ given in Equation (52) into the Lagrangian density Equation (51) will yield after tedious but straight-forward calculations the Lagrangian density:

(62) $$\begin{array}{ccc} L_{P} & \equiv & -\hat{\rho }\left[\frac{\partial \nu }{\partial t}+\alpha \frac{\partial \beta }{\partial t}+\varepsilon_{qt}+k\varphi +\frac{1}{2}{\left(\vec{\nabla }\nu +\alpha \vec{\nabla }\beta -k\vec{A}\right)}^{2}+\frac{\mu }{m}\vec{B}\cdot \hat{s}\right]\\ \varepsilon_{qt}\left[\hat{\rho },\alpha ,\beta \right] & \equiv & \varepsilon_{q}\left[\hat{\rho }\right]+\varepsilon_{qs}\left[\alpha ,\beta \right]\\ \varepsilon_{q}\left[\hat{\rho }\right] & \equiv & \frac{\hbar^{2}}{2m^{2}}\frac{{\left(\vec{\nabla }\sqrt{\hat{\rho }}\right)}^{2}}{\hat{\rho }}=\frac{\hbar^{2}}{2m^{2}}\frac{{\left(\vec{\nabla }R\right)}^{2}}{R^{2}}\\ \varepsilon_{qs}\left[\alpha ,\beta \right] & \equiv & \frac{\hbar^{2}}{8m^{2}}\left({\left(\vec{\nabla }\theta \right)}^{2}+\sin^{2}\theta {\left(\vec{\nabla }\phi \right)}^{2}\right)\\ & = & \frac{1}{2}\left({\left(\frac{\hbar }{2m}\right)}^{2}\frac{{\left(\vec{\nabla }\alpha \right)}^{2}}{1-\alpha^{2}}+\left(1-\alpha^{2}\right){\left(\vec{\nabla }\beta \right)}^{2}\right) \end{array}$$

The Lagrangian $L_{P}$ has the same form as the Clebsch Lagrangian $\hat{L}$ given in Equation (20). However, there are some important differences. The internal energy in the Pauli Lagrangian is positive as is the case for the barotropic fluid but now the internal energy depends on the derivatives of the degrees of freedom and not just on the density at a given point, in this sense this internal energy is non-local. Moreover, it is made of two parts the Schrödinger quantum internal energy $\varepsilon_{q}$ which depends on the mass density and the spin quantum internal energy $\varepsilon_{qs}$ that depend on the spin (vorticity) degrees of freedom. Finally, the classical limit ℏ→0 will eliminate $\varepsilon_{q}$ but will not eliminate the spin internal energy:

(63) $$\lim_{\hbar \to 0}\varepsilon_{qs}=\frac{1}{2}\left(1-\alpha^{2}\right){\left(\vec{\nabla }\beta \right)}^{2}$$

In this sense, the Pauli theory has no standard classical limit, although this limit is a perfectly legitimate classical field theory. The interaction terms of electron dipole moment with the magnetic field $\frac{\mu }{m}\vec{B}\cdot \hat{s}$ has no classical analogue either. Indeed, this term was introduced by Pauli on a empirical basis with no theoretical justification, that is Pauli was aiming to explain the Stern–Gerlach experiment. Later, however, it was obtained from the relativistic Dirac equation. This points to the conclusion that we have gone as far as possible in understanding quantum mechanics is a non-relativistic framework. Additionally, further insight can only be gained by considering relativistic fluids, which are unfortunately beyond the scope of the current paper. This is indeed peculiar, as sometimes it is claimed that quantum mechanics and relativity (mainly general relativity) are contradictory when it now seems to be that quantum mechanics cannot be understood without relativity.

We conclude this section by writing down the equations for the Clebsch–Pauli variables, unfortunately those equations are not very helpful. Taking the variational derivative we arrive at the equations of motion (see also [3] p. 392, Equations (9.3.6), (9.3.17) and (9.3.18)):

(64) $$\begin{array}{ccc} & & \frac{\partial \hat{\rho }}{\partial t}+\vec{\nabla }\cdot \left(\hat{\rho }\vec{v}\right)=0\\ & & \frac{d\alpha }{dt}=\frac{1}{\hat{\rho }}\vec{\nabla }\cdot \left(\hat{\rho }\left(\alpha^{2}-1\right)\vec{\nabla }\beta \right)+\frac{2\mu }{\hbar }{\left(\vec{B}\times \hat{s}\right)}_{3}\\ & & \frac{d\beta }{dt}={\left(\frac{\hbar }{2m}\right)}^{2}\frac{1}{\hat{\rho }\sqrt{1-\alpha^{2}}}\vec{\nabla }\cdot \left(\hat{\rho }\frac{\vec{\nabla }\alpha }{\sqrt{1-\alpha^{2}}}\right)+\alpha {\left(\vec{\nabla }\beta \right)}^{2}\\ & + & \frac{\mu }{m}\vec{B}\cdot \left(\cot\theta \sin\Phi ,\cot\theta \cos\Phi ,-1\right)\\ & & \frac{d\nu }{dt}=\frac{1}{2}{\vec{v}}^{2}-e\varphi -\alpha^{2}{\left(\vec{\nabla }\beta \right)}^{2}-\frac{Q}{m}-\varepsilon_{qs}\\ & & -{\left(\frac{\hbar }{2m}\right)}^{2}\frac{\alpha }{\hat{\rho }\sqrt{1-\alpha^{2}}}\vec{\nabla }\cdot \left(\hat{\rho }\frac{\vec{\nabla }\alpha }{\sqrt{1-\alpha^{2}}}\right)-\frac{\mu }{m}\vec{B}\cdot \hat{s}. \end{array}$$

We notice that, in spin fluid dynamics, α and β are not comoving scalar fields (labels) as in the case of ideal barotropic fluid dynamics. We are now in a position to calculate the material derivative of the velocity and obtain the spin fluid dynamics Euler equation ([3] p. 393, Equation (9.3.19)):

(65) $$\frac{d\vec{v}}{dt}=-\vec{\nabla }\left(\frac{Q}{m}\right)-{\left(\frac{\hbar }{2m}\right)}^{2}\frac{1}{\hat{\rho }}\partial_{k}\left(\hat{\rho }\vec{\nabla }{\hat{s}}_{j}\partial_{k}{\hat{s}}_{j}\right)+k\left(\vec{E}+\vec{v}\times \vec{B}\right)-\frac{\mu }{m}\left(\vec{\nabla }B_{j}\right)s_{j}$$

### Spin Flows in Terms of Fisher Information

As explained in the introduction, the quantum spin flow does not have a microstructure that will explain its internal energy, so it should not have an internal energy. Nevertheless, the Lagrangian density does contain a “quantum internal energy term”, as can be seen in Equation (62). The term can be written as:

(66) $$\hat{\rho }\varepsilon_{qt}=\hat{\rho }\varepsilon_{q}+\hat{\rho }\varepsilon_{qs}=\frac{\hbar^{2}}{8m}\left[4{\left(\vec{\nabla }R\right)}^{2}+R^{2}{\left(\vec{\nabla }\theta \right)}^{2}+R^{2}\sin^{2}\theta {\left(\vec{\nabla }\phi \right)}^{2}\right]$$

Using the amplitudes of Equation (55) and the definition of Fisher information density of Equation (25) we arrive at the result:

(67) $$F_{Ip}=F_{I↑}+F_{I↓}=4\left[{\left(\vec{\nabla }a_{↑}\right)}^{2}+{\left(\vec{\nabla }a_{↓}\right)}^{2}\right]=4{\left(\vec{\nabla }R\right)}^{2}+R^{2}{\left(\vec{\nabla }\theta \right)}^{2}$$

Hence:

(68) $$\hat{\rho }\varepsilon_{qt}=\frac{\hbar^{2}}{8m}F_{Ip}+\frac{1}{2}\hat{\rho }\left(1-\alpha^{2}\right){\left(\vec{\nabla }\beta \right)}^{2}$$

Thus, the Lagrangian density of the spin flow given in Equation (62) can be written as:

(69) $$L_{P}=-\hat{\rho }\left[\frac{\partial \nu }{\partial t}+\alpha \frac{\partial \beta }{\partial t}+\frac{1}{2}\left(1-\alpha^{2}\right){\left(\vec{\nabla }\beta \right)}^{2}+k\varphi +\frac{1}{2}{\left(\vec{\nabla }\nu +\alpha \vec{\nabla }\beta -k\vec{A}\right)}^{2}\right]-\frac{\hbar^{2}}{8m}F_{Ip}$$

The pre-factor $\frac{\hbar^{2}}{8m}$ seems to appear in every case in which Fisher information appears in a quantum Lagrangian and may be significant.

## 6. Conclusions

In the current work, which is a continuation of previous studies [24,38,40], we demonstrate how Pauli’s spinor can be interpreted in terms of spin fluid using a generalized Clebsch form which is modified to include the electromagnetic vector potential which should affect a charged fluid. The theory is described by an action and a variational principle, and the fluid equations are derived as the extrema of the action. The similarities, as well as the pronounced differences, with barotropic fluid dynamics are discussed.

A fundamental obstacle to the fluid interpretation of quantum mechanics still exist. This is related to the origin of thermodynamic quantities which are part of fluid mechanics in the quantum context. For classical fluid the thermodynamic internal energy implies that a fluid element is not a point particle but has internal structure. The standard thermodynamics notions such as specific enthalpy, pressure, and temperature are derived from the specific internal energy equation of state which in turn assumes the said internal structure. The internal energy is a required component of any Lagrangian density attempting to depict a fluid. The unique form of the internal energy can be derived in principle relying on the basis of the atoms and molecules from which the fluid is composed and their interactions using statistical physics. However, the quantum fluid has no such microscopic structure and yet analysis of both the spinless [5,6] and spin [24] quantum fluid shows that terms analogue to internal energies appear. Thus, one is forced to ask where do those internal energies originate, surely the quantum fluid is devoid of a microscopic substructure as this will defy the empirically supported conception of the electron as a point particle. The answer to this inquiry originated from measurement theory [37]. Fisher information a basic concept of measurement theory is a measure of the quality of the measurement. It was shown that this concept is proportional to the internal energy of Schrödinger’s spinless electron which is essentially a theory of a potential flow which moves under the influence of electromagnetic fields and Fisher information forces. Fisher information can also explain most parts of the internal energy of an electron with spin. This puts (Fisher) information as a fundamental force of nature, which has the same status as electromagnetic forces in the quantum mechanical level of reality. Indeed, according to Anton Zeilinger’s recent remark to the press, it is quantum mechanics that demonstrates that information is more fundamental than space-time.

We have highlighted the similarities between the variational principles of Eulerian fluid mechanics and both Schrödinger’s and Pauli’s quantum mechanics as opposed to classical mechanics. The former have only linear time derivatives of degrees of freedom while that later have quadratic time derivatives. The former contain terms quadratic in the vector potential $\vec{A}$ while the later contain only linear terms, thus making the mass current and the current which couple to vector potential proportional. The former are manifestly gauge invariant which is achieved by redefining a phase or a flow potential.

To conclude we suggest the following future directions of research:

1. It is conjectured that same analogy found between Pauli’s theory and fluid dynamics may be found between Dirac’s relativistic electron theory and relativistic fluid dynamics.
2. It is also conjectured that an Eulerian relativistic fluid once properly defined in terms of a variational principle and augmented by a four dimensional Fisher information term will fully explain Dirac’s relativistic electron theory and inter alia Pauli’s theory as well.

## Data Availability

Not applicable.

## Appendix A. A Classical Charged Particle

Consider a classical particle with the coordinates $\vec{x}\left(t\right)$, mass *m* and charge *e* interacting with a given electromagnetic vector potential $\vec{A}\left(\vec{x},t\right)$ and scalar potential $\varphi (\vec{x},t)$. We will not be interested in the effects of the particle on the field and thus consider the field as “external”. The action of said particle is:

(A1) $$\begin{array}{ccc} A & = & \int_{t1}^{t2}Ldt,\;L=L_{0}+L_{i}\\ L_{0} & \equiv & \frac{1}{2}mv^{2},\;L_{i}\equiv e\left(\vec{A}\cdot \vec{v}-\varphi \right),\;\vec{v}\equiv \frac{d\vec{x}}{dt}\equiv \dot{\vec{x}},\;v=\left|\vec{v}\right|. \end{array}$$

The variation of the two parts of the Lagrangian are given by:

(A2) $$\delta L_{0}=m\dot{\vec{x}}\cdot \delta \dot{\vec{x}}=\frac{d(m\vec{v}\cdot \delta \vec{x})}{dt}-m\dot{\vec{v}}\cdot \delta \vec{x}$$

(A3) $$\begin{array}{ccc} \delta L_{i} & = & e\left(\delta \vec{A}\cdot \vec{v}+\vec{A}\cdot \delta \dot{\vec{x}}-\delta \varphi \right)\\ & = & e\left(\partial_{k}\vec{A}\cdot \vec{v}\delta x_{k}+\frac{d(\vec{A}\cdot \delta \vec{x})}{dt}-\delta \vec{x}\cdot \frac{d\vec{A}}{dt}-\vec{\nabla }\varphi \cdot \delta \vec{x}\right), \end{array}$$

in the above $\partial_{k}\equiv \frac{\partial }{\partial x_{k}}$ and $\vec{\nabla }\equiv \left(\frac{\partial }{\partial x},\frac{\partial }{\partial y},\frac{\partial }{\partial z}\right)\equiv \left(\frac{\partial }{\partial x_{1}},\frac{\partial }{\partial x_{2}},\frac{\partial }{\partial x_{3}}\right)$. We use the Einstein summation convention in which a Latin index (say k,l) takes one of the values k,l∈[1,2,3]. We may write the total time derivative of $\vec{A}$ as:

(A4) $$\frac{d\vec{A}\left(\vec{x}\left(t\right),t\right)}{dt}=\partial_{t}\vec{A}+v_{l}\partial_{l}\vec{A},\;\partial_{t}\equiv \frac{\partial }{\partial t}.$$

Thus, the variation $\delta L_{i}$ can be written in the following form:

(A5) $$\delta L_{i}=\frac{d(e\vec{A}\cdot \delta \vec{x})}{dt}+e\left[\left(\partial_{k}A_{l}-\partial_{l}A_{k}\right)v_{l}-\partial_{t}A_{k}-\partial_{k}\varphi \right]\delta x_{k},$$

Defining the electric and magnetic fields in the standard way:

(A6) $$\vec{B}\equiv \vec{\nabla }\times \vec{A},\;\vec{E}\equiv -\partial_{t}\vec{A}-\vec{\nabla }\varphi ,$$

it follows that:

(A7) $$\epsilon_{kln}B_{n}=\partial_{k}A_{l}-\partial_{l}A_{k},\;E_{k}=-\partial_{t}A_{k}-\partial_{k}\varphi ,$$

in which $\epsilon_{kln}$ is the three index antisymmetric tensor. Thus, we may write $\delta L_{i}$ as:

(A8) $$\delta L_{i}=\frac{d(e\vec{A}\cdot \delta \vec{x})}{dt}+e\left[\epsilon_{kln}v_{l}B_{n}+E_{k}\right]\delta x_{k}=\frac{d(e\vec{A}\cdot \delta \vec{x})}{dt}+e\left[\vec{v}\times \vec{B}+\vec{E}\right]\cdot \delta \vec{x}.$$

We use the standard definition of the Lorentz force (MKS units):

(A9) $${\vec{F}}_{L}\equiv e\left[\vec{v}\times \vec{B}+\vec{E}\right]$$

to write:

(A10) $$\delta L_{i}=\frac{d(e\vec{A}\cdot \delta \vec{x})}{dt}+{\vec{F}}_{L}\cdot \delta \vec{x}.$$

Combining the variation of $L_{i}$ given in Equation (A10) and the variation of $L_{0}$ given in Equation (A2), it follows from Equation (A1) that the variation of *L* is:

(A11) $$\delta L=\delta L_{0}+\delta L_{i}=\frac{d\left(\left(m\vec{v}+e\vec{A}\right)\cdot \delta \vec{x}\right)}{dt}+\left(-m\dot{\vec{v}}+{\vec{F}}_{L}\right)\cdot \delta \vec{x}.$$

Thus, the variation of the action is:

(A12) $$\delta A=\int_{t1}^{t2}\delta Ldt={\left.\left(m\vec{v}+e\vec{A}\right)\cdot \delta \vec{x}\right|}_{t1}^{t2}-\int_{t1}^{t2}\left(m\dot{\vec{v}}-{\vec{F}}_{L}\right)\cdot \delta \vec{x}dt.$$

Since the classical trajectory is such that the variation of the action on it vanishes for a small modification of the trajectory $\delta \vec{x}$ that vanishes at t1 and t2 but is otherwise arbitrary it follows that:

(A13) $$m\dot{\vec{v}}={\vec{F}}_{L}=e\left[\vec{v}\times \vec{B}+\vec{E}\right]\Rightarrow \dot{\vec{v}}=\frac{e}{m}\left[\vec{v}\times \vec{B}+\vec{E}\right].$$

Thus, the dynamics of a classical particle in a given electric and magnetic field is described by a single number, the ratio between its charge and mass:

(A14) $$k\equiv \frac{e}{m}\;\Rightarrow \;\dot{\vec{v}}=k\left[\vec{v}\times \vec{B}+\vec{E}\right].$$

The reader is reminded that the connection between the electromagnetic potentials and the fields is not unique. Indeed performing a gauge transformation to obtain a new set of potentials:

(A15) $${\vec{A}}^{\prime }=\vec{A}+\vec{\nabla }\Lambda ,\;\varphi^{\prime }=\varphi -\partial_{t}\Lambda .$$

we obtain the same fields:

(A16) $${\vec{B}}^{\prime }=\vec{\nabla }\times {\vec{A}}^{\prime }=\vec{\nabla }\times \vec{A}=\vec{B},\;{\vec{E}}^{\prime }=-\partial_{t}{\vec{A}}^{\prime }-\vec{\nabla }\varphi^{\prime }=-\partial_{t}\vec{A}-\vec{\nabla }\varphi =\vec{E}.$$

For a system of *N* particles each with an index 1≤j≤N, a corresponding mass $m_{j}$, charge $e_{j}$, position vector ${\vec{x}}_{j}$, and velocity ${\vec{v}}_{j}\equiv \frac{d{\vec{x}}_{j}}{dt}$ the action and Lagrangian for each point particle are as follows:

(A17) $$\begin{array}{ccc} A_{j} & = & \int_{t1}^{t2}L_{j}dt,\;L_{j}=L_{0j}+L_{ij}\\ L_{0j} & \equiv & \frac{1}{2}m_{j}v_{j}^{2},\;L_{ij}\equiv e_{j}\left(\vec{A}\left({\vec{x}}_{j},t\right)\cdot {\vec{v}}_{j}-\varphi \left({\vec{x}}_{j},t\right)\right). \end{array}$$

The action and Lagrangian of the system of particles is:

(A18) $$A_{s}=\int_{t1}^{t2}L_{s}dt,\;L_{s}=\sum_{j=1}^{N}L_{j}.$$

The variational analysis follows the same lines as for a single particle and we obtain a set of equations of the form:

(A19) $${\dot{\vec{v}}}_{j}=\frac{e_{j}}{m_{j}}\left[{\vec{v}}_{j}\times \vec{B}\left({\vec{x}}_{j},t\right)+\vec{E}\left({\vec{x}}_{j},t\right)\right],\;j\in \left[1-N\right].$$

## Appendix B. A Classical Charged Fluid—The Lagrangian Approach

The dynamics of the fluid is determined by its composition and the forces acting on it. The fluid is made of “fluid elements” [29,44], practically a “fluid element” is a point particle which has an infinitesimal mass $dM_{\vec{\alpha }}$, infinitesimal charge $dQ_{\vec{\alpha }}$, position vector $\vec{x}\left(\vec{\alpha },t\right)$, and velocity $\vec{v}\left(\vec{\alpha },t\right)\equiv \frac{d\vec{x}\left(\vec{\alpha },t\right)}{dt}$. Here, the continuous vector label $\vec{\alpha }$ replaces the discrete index *j* of the previous section. As the “fluid element” is not truly a point particle, it has, also, an infinitesimal volume $dV_{\vec{\alpha }}$, infinitesimal entropy $dS_{\vec{\alpha }}$, and an infinitesimal internal energy $dE_{in\;\vec{\alpha }}$. The action and Lagrangian for each “fluid element” are according to Equation (A1) as follows:

(A20) $$\begin{array}{ccc} dA_{\vec{\alpha }} & = & \int_{t1}^{t2}dL_{\vec{\alpha }}dt,\;dL_{\vec{\alpha }}=dL_{0\vec{\alpha }}+dL_{i\vec{\alpha }}\\ dL_{0\vec{\alpha }} & \equiv & \frac{1}{2}dM_{\vec{\alpha }}\;v{\left(\vec{\alpha },t\right)}^{2}-dE_{in\;\vec{\alpha }},\\ dL_{i\vec{\alpha }} & \equiv & dQ_{\vec{\alpha }}\left(\vec{A}\left(\vec{x}\left(\vec{\alpha },t\right),t\right)\cdot \vec{v}\left(\vec{\alpha },t\right)-\varphi \left(\vec{x}\left(\vec{\alpha },t\right),t\right)\right). \end{array}$$

All the above quantities are calculated for a specific value of the label $\vec{\alpha }$, while the action and Lagrangian of the entire fluid, should be summed (or integrated) over all possible $\vec{\alpha }$’s. That is:

(A21) $$\begin{array}{ccc} L & = & \int_{\vec{\alpha }}dL_{\vec{\alpha }}\\ A & = & \int_{\vec{\alpha }}dA_{\vec{\alpha }}=\int_{t1}^{t2}\int_{\vec{\alpha }}dL_{\vec{\alpha }}dt=\int_{t1}^{t2}Ldt. \end{array}$$

It is customary to define densities for the Lagrangian, mass, and charge of every fluid element as follows:

(A22) $$L_{\vec{\alpha }}\equiv \frac{dL_{\vec{\alpha }}}{dV_{\vec{\alpha }}},\;\rho_{\vec{\alpha }}\equiv \frac{dM_{\vec{\alpha }}}{dV_{\vec{\alpha }}},\;\rho_{c\vec{\alpha }}\equiv \frac{dQ_{\vec{\alpha }}}{dV_{\vec{\alpha }}}$$

Each of the above quantities may be thought of as a function of the location $\vec{x}$, where the “fluid element” labelled $\vec{\alpha }$ happens to be in time *t*, for example:

(A23) $$\rho \left(\vec{x},t\right)\equiv \rho \left(\vec{x}\left(\vec{\alpha },t\right),t\right)\equiv \rho_{\vec{\alpha }}\left(t\right)$$

It is also customary to define the specific internal energy $\varepsilon_{\vec{\alpha }}$ as follows:

(A24) $$\varepsilon_{\vec{\alpha }}\equiv \frac{dE_{in\;\vec{\alpha }}}{dM_{\vec{\alpha }}}\;\Rightarrow \;\rho_{\vec{\alpha }}\varepsilon_{\vec{\alpha }}=\frac{dM_{\vec{\alpha }}}{dV_{\vec{\alpha }}}\frac{dE_{in\;\vec{\alpha }}}{dM_{\vec{\alpha }}}=\frac{dE_{in\;\vec{\alpha }}}{dV_{\vec{\alpha }}}$$

Thus, we can write the following equations for the Lagrangian density:

(A25) $$\begin{array}{ccc} L_{\vec{\alpha }} & = & \frac{dL_{\vec{\alpha }}}{dV_{\vec{\alpha }}}=\frac{{dL}_{0\vec{\alpha }}}{dV_{\vec{\alpha }}}+\frac{{dL}_{i\vec{\alpha }}}{dV_{\vec{\alpha }}}=L_{0\vec{\alpha }}+L_{i\vec{\alpha }}\\ L_{0\vec{\alpha }} & \equiv & \frac{1}{2}\rho_{\vec{\alpha }}v{\left(\vec{\alpha },t\right)}^{2}-\rho_{\vec{\alpha }}\varepsilon_{\vec{\alpha }},\\ L_{i\vec{\alpha }} & \equiv & \rho_{c\vec{\alpha }}\left(\vec{A}\left(\vec{x}\left(\vec{\alpha },t\right),t\right)\cdot \vec{v}\left(\vec{\alpha },t\right)-\varphi \left(\vec{x}\left(\vec{\alpha },t\right),t\right)\right). \end{array}$$

The above expression allows us to write the Lagrangian as a spatial integral:

(A26) $$L=\int_{\vec{\alpha }}dL_{\vec{\alpha }}=\int_{\vec{\alpha }}L_{\vec{\alpha }}dV_{\vec{\alpha }}=\int L\left(\vec{x},t\right)d^{3}x$$

which will be important for later sections of the current paper. Returning now to the variational analysis we introduce the symbols $\Delta {\vec{x}}_{\vec{\alpha }}\equiv {\vec{\xi }}_{\vec{\alpha }}$ to indicate a variation of the trajectory ${\vec{x}}_{\vec{\alpha }}\left(t\right)$ (we reserve the symbol δ in the fluid context, to a different kind of variation, the Eulerian variation). In an ideal fluid the “fluid element” does not exchange mass, nor electric charge, nor heat with other fluid elements, so it follows that:

(A27) $$\Delta dM_{\vec{\alpha }}=\Delta dQ_{\vec{\alpha }}=\Delta dS_{\vec{\alpha }}=0.$$

Moreover, according to thermodynamics a change in the internal energy of a “fluid element” satisfies the equation:

(A28) $$\Delta dE_{in\;\vec{\alpha }}=T_{\vec{\alpha }}\Delta dS_{\vec{\alpha }}-P_{\vec{\alpha }}\Delta dV_{\vec{\alpha }},$$

The first term describes the heating energy gained by the “fluid element”, while the second terms describe the work performed by the “fluid element” on neighbouring elements. $T_{\vec{\alpha }}$ is the temperature of the “fluid element” and $P_{\vec{\alpha }}$ is the pressure of the same. As the mass of the fluid element does not change, we may divide the above expression by $dM_{\vec{\alpha }}$ to obtain the variation of the specific energy as follows:

(A29) $$\begin{array}{ccc} \Delta \varepsilon_{\vec{\alpha }} & = & \Delta \frac{dE_{in\;\vec{\alpha }}}{dM_{\vec{\alpha }}}=T_{\vec{\alpha }}\Delta \frac{dS_{\vec{\alpha }}}{dM_{\vec{\alpha }}}-P_{\vec{\alpha }}\Delta \frac{dV_{\vec{\alpha }}}{dM_{\vec{\alpha }}}\\ & = & T_{\vec{\alpha }}\Delta s_{\vec{\alpha }}-P_{\vec{\alpha }}\Delta \frac{1}{\rho_{\vec{\alpha }}}=T_{\vec{\alpha }}\Delta s_{\vec{\alpha }}+\frac{P_{\vec{\alpha }}}{\rho_{\vec{\alpha }}^{2}}\Delta \rho_{\vec{\alpha }}.\;s_{\vec{\alpha }}\equiv \frac{dS_{\vec{\alpha }}}{dM_{\vec{\alpha }}} \end{array}$$

in which $s_{\vec{\alpha }}$ is the specific entropy of the fluid element. It follows that:

(A30) $$\frac{\partial \varepsilon }{\partial s}=T,\;\frac{\partial \varepsilon }{\partial \rho }=\frac{P}{\rho^{2}}.$$

Another important thermodynamic quantity that we will use later is the enthalpy defined for a fluid element as:

(A31) $$dW_{\vec{\alpha }}=dE_{in\;\vec{\alpha }}+P_{\vec{\alpha }}dV_{\vec{\alpha }}.$$

and the specific enthalpy:

(A32) $$w_{\vec{\alpha }}=\frac{dW_{\vec{\alpha }}}{dM_{\vec{\alpha }}}=\frac{dE_{in\;\vec{\alpha }}}{dM_{\vec{\alpha }}}+P_{\vec{\alpha }}\frac{dV_{\vec{\alpha }}}{dM_{\vec{\alpha }}}=\varepsilon_{\vec{\alpha }}+\frac{P_{\vec{\alpha }}}{\rho_{\vec{\alpha }}}.$$

Combining the above result with Equation (A30) it follows that:

(A33) $$w=\varepsilon +\frac{P}{\rho }=\varepsilon +\rho \frac{\partial \varepsilon }{\partial \rho }=\frac{\partial (\rho \varepsilon )}{\partial \rho }.$$

Moreover:

(A34) $$\frac{\partial w}{\partial \rho }=\frac{\partial (\varepsilon +\frac{P}{\rho })}{\partial \rho }=-\frac{P}{\rho^{2}}+\frac{1}{\rho }\frac{\partial P}{\partial \rho }+\frac{\partial \varepsilon }{\partial \rho }=-\frac{P}{\rho^{2}}+\frac{1}{\rho }\frac{\partial P}{\partial \rho }+\frac{P}{\rho^{2}}=\frac{1}{\rho }\frac{\partial P}{\partial \rho }.$$

As we assume an ideal fluid, there is no heat conduction or heat radiation, and thus heat can only be moved around along the trajectory of the “fluid elements”, that is only convection is taken into account. Thus, $\Delta dS_{\vec{\alpha }}=0$ and we have:

(A35) $$\Delta dE_{in\;\vec{\alpha }}=-P\Delta dV_{\vec{\alpha }}.$$

Our next step would to be to evaluate the variation of the volume element. Suppose a time *t* the volume of the fluid element labelled by $\vec{\alpha }$ is described as:

(A36) $$dV_{\vec{\alpha },t}=d^{3}x\left(\vec{\alpha },t\right)$$

Using the Jacobian determinant we may relate this to the same element at t=0:

(A37) $$d^{3}x\left(\vec{\alpha },t\right)=Jd^{3}x\left(\vec{\alpha },0\right),\;J\equiv {\vec{\nabla }}_{0}x_{1}\cdot \left({\vec{\nabla }}_{0}x_{2}\times {\vec{\nabla }}_{0}x_{3}\right)$$

In which ${\vec{\nabla }}_{0}$ is taken with respect to the coordinates of the fluid elements at t=0: ${\vec{\nabla }}_{0}\equiv \left(\frac{\partial }{\partial x{\left(\vec{\alpha },0\right)}_{1}},\frac{\partial }{\partial x{\left(\vec{\alpha },0\right)}_{2}},\frac{\partial }{\partial x{\left(\vec{\alpha },0\right)}_{3}}\right)$. As both the actual and varied “fluid element” trajectories start at the same point it follows that:

(A38) $$\begin{array}{ccc} \Delta dV_{\vec{\alpha },t} & = & \Delta d^{3}x\left(\vec{\alpha },t\right)=\Delta J\;d^{3}x\left(\vec{\alpha },0\right)=\frac{\Delta J}{J}d^{3}x\left(\vec{\alpha },t\right)=\frac{\Delta J}{J}dV_{\vec{\alpha },t},\\ (\Delta d^{3}x\left(\vec{\alpha },0\right) & = & 0). \end{array}$$

The variation of *J* can be easily calculated as:

(A39) $$\Delta J={\vec{\nabla }}_{0}\Delta x_{1}\cdot \left({\vec{\nabla }}_{0}x_{2}\times {\vec{\nabla }}_{0}x_{3}\right)+{\vec{\nabla }}_{0}x_{1}\cdot \left({\vec{\nabla }}_{0}\Delta x_{2}\times {\vec{\nabla }}_{0}x_{3}\right)+{\vec{\nabla }}_{0}x_{1}\cdot \left({\vec{\nabla }}_{0}x_{2}\times {\vec{\nabla }}_{0}\Delta x_{3}\right),$$

Now:

(A40) $$\begin{array}{ccc} & & {\vec{\nabla }}_{0}\Delta x_{1}\cdot \left({\vec{\nabla }}_{0}x_{2}\times {\vec{\nabla }}_{0}x_{3}\right)={\vec{\nabla }}_{0}\xi_{1}\cdot \left({\vec{\nabla }}_{0}x_{2}\times {\vec{\nabla }}_{0}x_{3}\right)\\ & = & \partial_{k}\xi_{1}{\vec{\nabla }}_{0}x_{k}\cdot \left({\vec{\nabla }}_{0}x_{2}\times {\vec{\nabla }}_{0}x_{3}\right)=\partial_{1}\xi_{1}{\vec{\nabla }}_{0}x_{1}\cdot \left({\vec{\nabla }}_{0}x_{2}\times {\vec{\nabla }}_{0}x_{3}\right)=\partial_{1}\xi_{1}J.\\ & & {\vec{\nabla }}_{0}x_{1}\cdot \left({\vec{\nabla }}_{0}\Delta x_{2}\times {\vec{\nabla }}_{0}x_{3}\right)={\vec{\nabla }}_{0}x_{1}\cdot \left({\vec{\nabla }}_{0}\xi_{2}\times {\vec{\nabla }}_{0}x_{3}\right)\\ & = & \partial_{k}\xi_{2}{\vec{\nabla }}_{0}x_{1}\cdot \left({\vec{\nabla }}_{0}x_{k}\times {\vec{\nabla }}_{0}x_{3}\right)=\partial_{2}\xi_{2}{\vec{\nabla }}_{0}x_{1}\cdot \left({\vec{\nabla }}_{0}x_{2}\times {\vec{\nabla }}_{0}x_{3}\right)=\partial_{2}\xi_{2}J.\\ & & {\vec{\nabla }}_{0}x_{1}\cdot \left({\vec{\nabla }}_{0}x_{2}\times {\vec{\nabla }}_{0}\Delta x_{3}\right)={\vec{\nabla }}_{0}x_{1}\cdot \left({\vec{\nabla }}_{0}x_{2}\times {\vec{\nabla }}_{0}\xi_{3}\right)\\ & = & \partial_{k}\xi_{3}{\vec{\nabla }}_{0}x_{1}\cdot \left({\vec{\nabla }}_{0}x_{2}\times {\vec{\nabla }}_{0}x_{k}\right)=\partial_{3}\xi_{3}{\vec{\nabla }}_{0}x_{1}\cdot \left({\vec{\nabla }}_{0}x_{2}\times {\vec{\nabla }}_{0}x_{3}\right)=\partial_{3}\xi_{3}J. \end{array}$$

Combining the above results, it follows that:

(A41) $$\Delta J=\partial_{1}\xi_{1}J+\partial_{2}\xi_{2}J+\partial_{3}\xi_{3}J=\vec{\nabla }\cdot \vec{\xi }\;J.$$

Which allows us to calculate the variation of the volume of the “fluid element”:

(A42) $$\Delta dV_{\vec{\alpha },t}=\vec{\nabla }\cdot \vec{\xi }\;dV_{\vec{\alpha },t}.$$

Thus the variation of the internal energy is:

(A43) $$\Delta dE_{in\;\vec{\alpha }}=-P\vec{\nabla }\cdot \vec{\xi }\;dV_{\vec{\alpha },t}.$$

The internal energy is the only novel element with respect to the single particle scenario and system of particles scenario described in the previous section, thus the rest of the variation analysis is straight forward. Varying Equation (A20) we obtain:

(A44) $$\begin{array}{ccc} \Delta dA_{\vec{\alpha }} & = & \int_{t1}^{t2}\Delta dL_{\vec{\alpha }}dt,\;\Delta dL_{\vec{\alpha }}=\Delta dL_{0\vec{\alpha }}+\Delta dL_{i\vec{\alpha }}\\ \Delta dL_{0\vec{\alpha }} & = & dM_{\vec{\alpha }}\;\vec{v}\left(\vec{\alpha },t\right)\cdot \Delta \vec{v}\left(\vec{\alpha },t\right)-\Delta dE_{in\;\vec{\alpha }},\\ \Delta dL_{i\vec{\alpha }} & = & dQ_{\vec{\alpha }}\left(\Delta \vec{A}\left(\vec{x}\left(\vec{\alpha },t\right),t\right)\cdot \vec{v}\left(\vec{\alpha },t\right)+\vec{A}\left(\vec{x}\left(\vec{\alpha },t\right),t\right)\cdot \Delta \vec{v}\left(\vec{\alpha },t\right)\right.\\ & - & \left.\Delta \varphi (\vec{x}\left(\vec{\alpha }\right),t)\right). \end{array}$$

Notice that:

(A45) $$\Delta \vec{v}\left(\vec{\alpha },t\right)=\Delta \frac{d\vec{x}\left(\vec{\alpha },t\right)}{dt}=\frac{d\Delta \vec{x}\left(\vec{\alpha },t\right)}{dt}=\frac{d\vec{\xi }\left(\vec{\alpha },t\right)}{dt}.$$

After some steps, which are described prior to Equation (A2), we obtain the variation of $dL_{0\vec{\alpha }}$:

(A46) $$\Delta dL_{0\vec{\alpha }}=\frac{d(dM_{\vec{\alpha }}{\vec{v}}_{\vec{\alpha }}\cdot {\vec{\xi }}_{\vec{\alpha }})}{dt}-dM_{\vec{\alpha }}\frac{d{\vec{v}}_{\vec{\alpha }}}{dt}\cdot {\vec{\xi }}_{\vec{\alpha }}+P\vec{\nabla }\cdot {\vec{\xi }}_{\vec{\alpha }}\;dV_{\vec{\alpha },t}.$$

Similarly, the analogue equations of Equation (A9) and Equation (A10) are:

(A47) $$d{\vec{F}}_{L\vec{\alpha }}\equiv dQ_{\vec{\alpha }}\left[{\vec{v}}_{\vec{\alpha }}\times \vec{B}\left(\vec{x}\left(\vec{\alpha },t\right),t\right)+\vec{E}\left(\vec{x}\left(\vec{\alpha },t\right),t\right)\right]$$

and:

(A48) $$\Delta dL_{i\vec{\alpha }}=\frac{d(dQ_{\vec{\alpha }}\vec{A}\left(\vec{x}\left(\vec{\alpha },t\right),t\right)\cdot {\vec{\xi }}_{\vec{\alpha }})}{dt}+d{\vec{F}}_{L\vec{\alpha }}\cdot {\vec{\xi }}_{\vec{\alpha }}.$$

The variation of the action of a single fluid element is thus:

(A49) $$\begin{array}{ccc} \Delta dA_{\vec{\alpha }} & = & \int_{t1}^{t2}\Delta dL_{\vec{\alpha }}dt={\left.\left(dM_{\vec{\alpha }}\vec{v}\left(\vec{\alpha },t\right)+dQ_{\vec{\alpha }}\vec{A}\left(\vec{x}\left(\vec{\alpha },t\right),t\right)\right)\cdot {\vec{\xi }}_{\vec{\alpha }}\right|}_{t1}^{t2}\\ & - & \int_{t1}^{t2}\left(dM_{\vec{\alpha }}\frac{d\vec{v}\left(\vec{\alpha },t\right)}{dt}\cdot {\vec{\xi }}_{\vec{\alpha }}-d{\vec{F}}_{L\vec{\alpha }}\cdot {\vec{\xi }}_{\vec{\alpha }}-P\vec{\nabla }\cdot {\vec{\xi }}_{\vec{\alpha }}\;dV_{\vec{\alpha },t}\right)dt. \end{array}$$

The variation of the total action of the fluid is thus:

(A50) $$\begin{array}{ccc} \Delta A & = & \int_{\vec{\alpha }}dA_{\vec{\alpha }}={\left.\int_{\vec{\alpha }}\left(dM_{\vec{\alpha }}\vec{v}\left(\vec{\alpha },t\right)+dQ_{\vec{\alpha }}\vec{A}\left(\vec{x}\left(\vec{\alpha },t\right),t\right)\right)\cdot {\vec{\xi }}_{\vec{\alpha }}\right|}_{t1}^{t2}\\ & - & \int_{t1}^{t2}\int_{\vec{\alpha }}\left(dM_{\vec{\alpha }}\frac{d\vec{v}\left(\vec{\alpha },t\right)}{dt}\cdot {\vec{\xi }}_{\vec{\alpha }}-d{\vec{F}}_{L\vec{\alpha }}\cdot {\vec{\xi }}_{\vec{\alpha }}-P\vec{\nabla }\cdot {\vec{\xi }}_{\vec{\alpha }}\;dV_{\vec{\alpha }}\right)dt. \end{array}$$

Now according to Equation (A22) we may write:

(A51) $$dM_{\vec{\alpha }}=\rho_{\vec{\alpha }}\;dV_{\vec{\alpha }},\;dQ_{\vec{\alpha }}=\rho_{c\vec{\alpha }}\;dV_{\vec{\alpha }}$$

using the above relations we may turn the $\vec{\alpha }$ integral into a volume integral and thus write the variation of the fluid action in which we suppress the $\vec{\alpha }$ labels:

(A52) $$\Delta A={\left.\int \left(\rho \vec{v}+\rho_{c}\vec{A}\right)\cdot \vec{\xi }dV\right|}_{t1}^{t2}-\int_{t1}^{t2}\int \left(\rho \frac{d\vec{v}}{dt}\cdot \vec{\xi }-{\vec{f}}_{L}\cdot \vec{\xi }-P\vec{\nabla }\cdot \vec{\xi }\right)dVdt.$$

in the above we introduced the Lorentz force density:

(A53) $${\vec{f}}_{L\vec{\alpha }}\equiv \frac{d{\vec{F}}_{L\vec{\alpha }}}{dV_{\vec{\alpha }}}=\rho_{c\vec{\alpha }}\left[{\vec{v}}_{\vec{\alpha }}\times \vec{B}\left(\vec{x}\left(\vec{\alpha },t\right),t\right)+\vec{E}\left(\vec{x}\left(\vec{\alpha },t\right),t\right)\right].$$

Now, since:

(A54) $$P\vec{\nabla }\cdot \vec{\xi }=\vec{\nabla }\cdot \left(P\vec{\xi }\right)-\vec{\xi }\cdot \vec{\nabla }P,$$

and using Gauss theorem the variation of the action can be written as:

(A55) $$\begin{array}{ccc} \Delta A & = & {\left.\int \left(\rho \vec{v}+\rho_{c}\vec{A}\right)\cdot \vec{\xi }dV\right|}_{t1}^{t2}\\ & - & \int_{t1}^{t2}\left[\int \left(\rho \frac{d\vec{v}}{dt}-{\vec{f}}_{L}+\vec{\nabla }P\right)\cdot \vec{\xi }dV-\oint P\vec{\xi }\cdot d\vec{\Sigma }\right]dt. \end{array}$$

It follows that the variation of the action will vanish for a $\vec{\xi }$, such that $\vec{\xi }\left(t1\right)=\vec{\xi }\left(t2\right)=0$ and vanishing on a surface encapsulating the fluid, but other than that arbitrary only if the Euler equation for a charged fluid is satisfied, that is:

(A56) $$\frac{d\vec{v}}{dt}=-\frac{\vec{\nabla }P}{\rho }+\frac{{\vec{f}}_{L}}{\rho },\;\frac{d}{dt}=\frac{\partial }{\partial t}+\vec{v}\cdot \vec{\nabla }$$

For the particular case that the fluid element is made of identical microscopic particles each with a mass *m* and a charge *e*, it follows that the mass and charge densities are proportional to the number density *n*:

(A57) $$\rho =m\;n,\;\rho_{c}=e\;n\Rightarrow \frac{{\vec{f}}_{L}}{\rho }=k\left[\vec{v}\times \vec{B}+\vec{E}\right],$$

Thus, except for the pressure terms, the equation is similar to that of a point particle. In experimental fluid dynamics, it is more convenient to describe a fluid in terms of quantities at a specific location, rather than quantities associated with unseen infinitesimal “fluid elements”. This road leads to the Eulerian description of fluid dynamics and thinking in terms of flow fields rather than in terms of a velocity of “fluid elements” as will be discussed in Section 2. This description will be shown later to be closely connected to quantum mechanics.
